# Antihypertensive medications and cancer risk: Evidence from 0.27 million patients with newly diagnosed hypertension

**DOI:** 10.3389/fphar.2025.1559604

**Published:** 2025-07-01

**Authors:** Huan Yu, Zhike Liu, Yiqun Wu, Liuyan Zheng, Kun Wang, Jingxian Wu, Huairong Wang, Kexin Ding, Ruotong Yang, Huziwei Zhou, Feng Sun, Yonghua Hu, Hongbo Lin, Peng Shen, Siyan Zhan

**Affiliations:** ^1^ Department of Epidemiology and Biostatistics, School of Public Health, Peking University Health Science Center, Beijing, China; ^2^ Key Laboratory of Epidemiology of Major Diseases (Peking University), Ministry of Education, Beijing, China; ^3^ Department of Epidemiology, Biostatistics and Occupational Health, McGill University, Montreal, QC, Canada; ^4^ Medical Informatics Center, Department of Epidemiology and Biostatistics, School of Public Health, Health Science Center, Peking University, Beijing, China; ^5^ Department of Data Center, Yinzhou District Center for Disease Control and Prevention, Ningbo, China; ^6^ Institute for Artificial Intelligence, Peking University, Beijing, China; ^7^ Research Center of Clinical Epidemiology, Peking University Third Hospital, Beijing, China

**Keywords:** hypertension, cancer, antihypertensive medications, real-world research, pharmacoepidemiology

## Abstract

**Background:**

Whether specific antihypertensive treatments increase cancer risk in patients with hypertension is still controversial. We aimed to estimate the associations of different antihypertensive treatments with cancer risk in real-world settings.

**Methods:**

A longitudinal cohort study was designed in a population of 1.2 million individuals from the *CHinese Electronic health Records Research in Yinzhou* (CHERRY). Propensity score matching (PSM) and the Cox regression model were used to estimate the associations. Several sensitivity analyses were then performed to reduce potential residual confounding.

**Results:**

From 2009 to 2019, a total of 270,320 patients with newly diagnosed hypertension were included in this study. With a median follow-up time of 7.7 years, 14,264 cases of cancer occurred. There were no significant associations of angiotensin-converting enzyme inhibitors (ACEIs), angiotensin receptor blockers (ARBs), β-blockers, or thiazide diuretics (TDs) with cancer risk (*p* > 0.05). Compared with other antihypertensive treatments, the use of calcium channel blockers (CCBs) was significantly associated with a marginally mild increase in the risk of all cancers (hazard ratio, HR = 1.05; 95% CI: 1.01, 1.09; *p* = 0.017). However, this association was no longer observed in sensitivity analyses excluding patients with less than 1, 2, or 3 years of follow-up. Nevertheless, the association between CCBs and the risk of endocrine cancer, especially thyroid cancer, still exists.

**Conclusion:**

Despite previous controversy, in this study, we found no clinically meaningful cancer risk associated with antihypertensive medications. However, the association of CCBs with specific cancer still requires further research. These findings should be interpreted with caution due to the potential residual confounding.

## Introduction

Hypertension is the leading preventable cause of cardiovascular disease (CVD), premature death, and disease burden globally (Zhou et al., 2021; Mills et al., 2020). Pharmacological therapy is one of the most important interventions for patients with hypertension to achieve optimal blood pressure (BP). Five first-line treatment drugs are recommended by the Chinese guidelines in 2018 and 2024 (Liu, 2020; Wang, 2025), namely, angiotensin-converting enzyme inhibitors (ACEIs), angiotensin receptor blockers (ARBs), beta-blockers, calcium channel blockers (CCBs), and thiazide diuretics (TDs).

The safety of antihypertensive drugs, including carcinogenic, has been widely concerned. Several studies showed a link between antihypertensive treatments and incidence of cancer, but their findings were conflicting. Two case–control studies suggested that the use of thiazides increased the risk of keratinocyte carcinoma (Pedersen et al., 2018; Pottegård et al., 2017), and a meta-analysis including 19 studies found that the use of CCBs and β-blockers was associated with a higher risk of skin cancer (Gandini et al., 2018). However, results from a recent cohort study did not find such an association (Drucker et al., 2021). Cell and animal studies found that CCBs can prevent pancreatic cancer development (Al-Wadei et al., 2009) and retard pancreatic cancer progression (Lin et al., 2012), whereas in another cohort study in postmenopausal women, a significantly increasing risk of pancreatic cancer was found in users of short-acting CCBs (Wang et al., 2018). A meta-analysis including two Asian studies found that ARB use reduced the risk of colorectal cancer among Asian populations (Qi et al., 2022), whereas no correlation was found between ARB use and colorectal cancer risk in an Asian population-based cohort study (Wang et al., 2023) and in another meta-analysis including 11 studies (Copland et al., 2021). In a case–control study in Spanish women, CCB was found to be associated with a higher risk of breast cancer, whereas ACEI/ARB drugs were not (Gómez-Acebo et al., 2016). However, a nested case–control study including 794,533 women demonstrated no association between any antihypertensive treatment and the risk of breast cancer (Chang et al., 2016). A meta-analysis including 21 studies also found no significant association between antihypertensive treatment use and breast cancer risk, but long-term use of ACEI/ARB might be beneficial for breast cancer prevention (Ni et al., 2017). In terms of lung cancer, a meta-analysis based on observational studies found an increased risk of lung cancer in ACEI users (Wu et al., 2023), but three other studies demonstrated that there were no association between ACEI use and lung cancer risk (Wang et al., 2023; Copland et al., 2021; Batais et al., 2021).

Evidence from previous studies is controversial and inconsistent (Tadic et al., 2019). An individual participant data-based meta-analysis including 33 RCTs was conceived to estimate the association between antihypertensive treatment and cancer risk. With a median follow-up time of 4.2 years, 15,012 cancer events were diagnosed among 260,447 participants. The research found no consistent evidence that antihypertensive medication use had any effect on cancer risk, but the potential risks of calcium channel blockers need to be noted (Copland et al., 2021). Given the limitations of the short follow-up time and heterogeneity among RCT studies, evidence from the real-world study is needed (Azoulay, 2021). Recently, a longitudinal study including 0.1 million participants with hypertension registered from community healthcare centers in China found that ARB/CCB use was associated with an increased risk of total cancer within a median follow-up time of 5.1 years (Wang et al., 2023), whereas, in the research, the absence of confounding control methods, such as propensity score matching (PSM), introduced a potential risk of bias. Consequently, the estimated associations diverged from the findings of the previously mentioned RCT meta-analysis (Azoulay, 2021).

Based on the above background and inconsistent evidence, we aimed to evaluate the association between antihypertensive treatment and cancer risk in a large Chinese cohort study. In this study, a population-based longitudinal study was designed from January 2009 to December 2022 in a real-world setting. The PSM approach and multiple sensitivity analyses were performed to reduce potential confounding between groups.

## Methods

### Study design, data sources, and populations

This research was designed as a longitudinal cohort study using real-world regional health information system data from the CHinese Electronic health Records Research in Yinzhou (CHERRY). The study is conducted in Yinzhou, Ningbo in Zhejiang Province, China, which is considered to have a completed electronic health information record system and has a national demonstration, with a total population of 1.2 million in 2016. The detailed protocols and more information have been described in previous studies (Liu et al., 2022; Lin et al., 2018).

The CHERRY study developed a comprehensive database including medical examination, regular epidemiological surveys, disease surveillance information, community health record, healthcare services, diagnosis of inpatient and outpatient, prescription, and medications, from local GPs, hospital systems, and pharmacies.

In this study, patients aged over 18 years with newly diagnosed hypertension from 1 January 2009 to 31 December 2019 were included and followed up until 1 January 2023. A washout window from 1 January 2005 to 31 December 2008 was established to recognize new cases of hypertension. Participants who met the following criteria were included in the study: (1) aged over 18 years on the index date, (2) with no hypertensive diagnosis or treatments within washout windows, (3) been diagnosed with hypertension using ICD-10 or with antihypertensive treatment during 2009–2019, and (4) had at least one prescription of five first-line antihypertensive treatments, namely, ACEI, ARB, β-blocker, CCB, and TD, since the diagnosis. Participants who had been diagnosed with cancer before the index date and who have used multiple antihypertensive treatment at the index date were excluded.

### Exposures

Of a population of 1.2 million, 270,320 patients with newly diagnosed hypertension who used antihypertensive treatment were included in this study. The index date was estimated when participants were first assigned a prescription for hypertensive medication. Exposure was determined based on the initial antihypertensive prescriptions at the index date, and then participants were divided into five groups: ACEI, ARB, β-blocker, CCB, and TD.

### Covariates

Multiple potential confounders were considered and controlled in this study. Demographics, including birthday, and sex, were collected at the time of registration, and age was then calculated at the index date. Habitual characteristics including drinking, smoking, and sports were obtained through regular epidemiological surveys. Physical measurements were conducted by GPs in primary care, and BMI and blood pressure were recorded. As comorbidities might influence outcomes, we used the Charlson Comorbidity Index (CCI) (detailed items and their corresponding weights are provided in Supplementary Table S1), along with dyslipidemia, depression, and arthritis to describe the comorbidity status in participants at the index date. CCI is a comprehensive quantized tool to evaluate comorbidity burden and predict risk of death (Charlson et al., 2022). Treatments of common chronic noncommunicable diseases including lipid-regulating agents, antidiabetic agents, nonsteroidal anti-inflammatory drugs (NSAIDs), corticoid agents, anticoagulants, and antiplatelet agents from the prescription database were also considered. The frequency of medical visits within 1 year before the index date was adjusted to control bias of individual differences in healthcare utilization. Participants may have been in different stages of the hypertension course when starting antihypertensive treatments, and the severity of hypertension may also have been different. In this study, we adjusted for the blood pressure level at the index date and the time from initial hypertension diagnosis to the initiation of antihypertensive medication to minimize potential confounding caused by the disease course. We also adjusted for adherence by categorizing participants into three groups according to the interquartile range of antihypertensive treatment frequency. Finally, the year of the index date was matched to reduce time-dependent bias.

### Follow-up and outcomes

Follow-up was conducted from the index date until the study outcome occurred, death occurred, or follow-up ended on 1 January 2023. Outpatient diagnosis information, discharge diagnosis information, and cancer report cards were used to ensure the incidence of cancer. Most diagnoses were linked to a national cancer registry, which performs annual pathological verification for a subset of cases, ensuring a high microscopic verification percentage (MV%). Death information and death time were collected using the death registration system.

The main outcome of this study was the incidence of all types of cancers. ICD-10 coding was used to identify the cancer types. Secondary outcomes included the occurrence of skin cancer, pancreatic cancer, colorectal cancer, breast cancer, and lung cancer, which have been controversial in previous studies. Cancer outcomes by location, coded using ICD-10, were also investigated, including lip, oral, and pharynx cancers (ICD-10: C00–C14); gastrointestinal cancer (C15–C26); respiratory neoplasms (C30–C39); skin cancer (C43–C44); female reproductive cancer (C51–C58); male reproductive cancer (C60–C63); urologic cancer (C64–C68); endocrine cancer (C73–C75); and leukemia (C81–C96).

### Statistical analysis

The characteristics of categorical variables were described using number and frequency. Normally distributed continuous variables were described using means and standard deviations, and non-normally distributed continuous variables were described using medians and quartiles.

To reduce potential confounding in real-world settings, the PSM method was performed to match age, sex, smoking status, drinking status, sports frequency, BMI, CCI, dyslipidemia history, depression history, arthritis history, lipid-regulating agent use, antidiabetic agent use, NSAID use, corticoid agent use, anticoagulant use, antiplatelet agent use, healthcare utilization, baseline blood pressure level, the time from initial hypertension diagnosis to the initiation of antihypertensive medication, adherence, and the year of the index date in any main comparison with a caliper width of 0.1. According to the sample size, 1:1, 1:1, 1:2, 1:4, and 1:4 ratios of matching were applied for CCB, ARB, β-blocker, TD, and ACEI, respectively. PSM was conducted after the inclusion and exclusion of research subjects. The Cox proportional hazard regression model was used to estimate the association between antihypertensive treatment and cancer risk in the matched paired dataset. Hazard ratio (HR) was used to describe the strength of association. To improve the power of the study, the inverse probability weighted (IPW) method considering all covariates was applied to determine the long-term effect of antihypertensive treatments on the risk of specific cancer types, and the group with other antihypertensive agents was defined as the control group.

To validate our results, we conducted subgroup analyses grouped by sex, age, and CCI and performed 10 sensitivity analyses: (1) only patients who persisted with single antihypertensive treatment without change during the follow-up period were included; (2) only patients with hypertension diagnosed based on ICD codes were included; (3) participants who started to use antihypertensive medication more than 1 year after the first diagnosis of hypertension were excluded; (4) an induction period was established to reduce reverse causality; participants who were followed up for less than 1 year/2 years/3 years were excluded to minimize the influence of reverse causation; (5) only the participants enrolled in this study from 2009 to 2017 to ensure a follow-up time of more than 5 years were included; (6) inverse probability weighting methods were used instead of PSM for association analysis. Probability was calculated using a logistic model adjusted for all covariates mentioned before; (7) competing risk models were used to account for the competing risk of death; (8) to address potential confounding from treatment modifications during follow-up, a time-dependent Cox proportional hazards model was used to evaluate the association between antihypertensive medication and cancer risk, which comprehensively incorporated all antihypertensive prescription records; (9) considering the confounding role of gynecological hormone therapy in breast cancer, we further adjusted for gynecological hormone therapy in the association analysis of breast cancer; (10) accounting for gastrointestinal cancer heterogeneity, we separately analyzed associations between antihypertensive drug and gastric and liver cancer, with additional adjustment for *Helicobacter pylori* infection in the gastric cancer analyses.

All analyses were performed using SAS 9.4 and R 4.3.1. The significance threshold (α) was set at 0.05.

## Results

A total of 270,320 participants with newly diagnosed hypertension who used antihypertensive treatment were enrolled in the study from 2009 to 2019. The mean age of participants was 55.4 (SD: 13.5) years, and 49.6% were female participants. Of all participants, 74.9% started antihypertensive treatment within 1 year after diagnosis of hypertension. In initial prescriptions, CCB accounted for the largest proportion of initial prescriptions for antihypertensive agents (39.8%), followed by ARBs (36.4%), β-blockers (13.4%), thiazide diuretics (6.2%), and ACEIs (4.3%). Baseline characteristics classed by initial antihypertensive agents are shown in Table 1 and Supplementary Table S2.

**TABLE 1:** Baseline characteristics of participants at the index date.

Characteristic	TD (N = 16,691)	ACEI (N = 11,502)	ARB (N = 98,278)	β-blocker (N = 36,162)	CCB (N = 107,687)	Overall (N = 270,320)
Age, mean (SD)	57.6 (14.4)	54.7 (13.6)	54.9 (12.9)	54.1 (15.3)	56.0 (13.1)	55.4 (13.5)
Female, N (%)	7,401 (44.3)	6,053 (52.6)	49,962 (50.8)	15,561 (43.0)	55,118 (51.2)	134,095 (49.6)
Baseline BP level, N (%)^a^						
<140/90	2024 (12.1)	1,326 (11.5)	11,022 (11.2)	2,384 (6.6)	11,984 (11.1)	28,740 (10.6)
140/90–160/100	1,233 (7.4)	777 (6.8)	6,510 (6.6)	1,364 (3.8)	7,187 (6.7)	17,071 (6.3)
160/100–180/110	332 (2.0)	228 (2.0)	2,103 (2.1)	388 (1.1)	2,468 (2.3)	5,519 (2.0)
>180/110	59 (0.4)	41 (0.4)	350 (0.4)	75 (0.2)	532 (0.5)	1,057 (0.4)
BMI, N (%)^a^						
<18.5	104 (0.6)	64 (0.6)	478 (0.5)	161 (0.4)	577 (0.5)	13,840.5)
18.5–25	2,396 (14.4)	1,571 (13.7)	13,855 (14.1)	2,921 (8.1)	15,263 (14.2)	36,006 (13.3)
25–30	1,365 (8.2)	963 (8.4)	7,759 (7.9)	1,487 (4.1)	8,538 (7.9)	20,112 (7.4)
>30	158 (0.9)	99 (0.9)	940 (1.0)	149 (0.4)	1,126 (1.0)	2,472 (0.9)
Smoking, N (%)	1,316 (7.9)	794 (6.9)	7,336 (7.5)	1975 (5.5)	8,207 (7.6)	19,628 (7.3)
Weekly drinking, N (%)	2085 (12.5)	1,505 (13.1)	13,637 (13.9)	4,688 (13.0)	14,999 (13.9)	36,914 (13.7)
CCI, mean (SD)	0.592 (1.06)	0.690 (1.17)	0.728 (1.17)	0.775 (1.25)	0.615 (1.07)	0.679 (1.14)
Weekly sports, N (%)	5,987 (35.9)	3,680 (32.0)	31,711 (32.3)	11,348 (31.4)	33,893 (31.5)	79,497 (31.5)
HTN course, median (IQR)	0 (0, 349)	1 (0, 471)	0 (0, 375)	0 (0, 121)	0 (0, 392)	0 (0, 313)
HCUI, median (IQR)	10 (4, 22)	8 (3, 18)	8 (3, 18)	9 (4, 21)	7 (3, 17)	8 (3, 18)
Dyslipidemia, N (%)	2030 (12.2)	1987 (17.3)	17,114 (17.4)	6,982 (19.3)	16,789 (15.6)	44,902 (16.6)
Depression, N (%)	119 (0.7)	118 (1.0)	1,045 (1.1)	879 (2.4)	1,029 (1.0)	3,190 (1.2)
Osteoarthritis, N (%)	4,165 (25.0)	2,502 (21.8)	25,173 (25.6)	9,639 (26.7)	26,189 (24.3)	67,668 (25.0)
Hypoglycemic therapy, N (%)	947 (5.7)	704 (6.1)	7,566 (7.7)	2,205 (6.1)	7,001 (6.5)	18,423 (6.8)
Lipid-lowering therapy, N (%)	715 (4.3)	547 (4.8)	5,644 (5.7)	1990 (5.5)	5,028 (4.7)	13,924 (5.2)
Corticoid therapy, N (%)	4,305 (25.8)	2,514 (21.9)	27,414 (27.9)	9,833 (27.2)	26,797 (24.9)	70,863 (26.2)
NSAIDs, N (%)	4,101 (24.6)	2,346 (20.4)	26,782 (27.3)	9,278 (25.7)	26,256 (24.4)	68,763 (25.4)
Adherence, N (%)						
Low	4,148 (24.9)	2,818 (24.5)	23,751 (24.2)	8,789 (24.3)	25,922 (24.1)	65,428 (24.2)
Middle	8,039 (48.2)	5,588 (48.6)	48,612 (49.5)	17,580 (48.6)	53,246 (49.4)	133,065 (49.2)
High	4,504 (27.0)	3,096 (26.9)	25,915 (26.4)	9,793 (27.1)	28,519 (26.5)	71,827 (26.6)

TD, thiazide diuretic; ACEI, angiotensin-converting enzyme inhibitor; ARB, angiotensin receptor blocker; CCB, calcium channel blocker; CCI, Charlson Comorbidity Index; HTN, hypertension; HCUI, Healthcare Utilization Index; NSAIDs, nonsteroidal anti-inflammatory drugs.

^a^ The discrepancy between the sum and total sample size is attributed to missing data.

With a median follow-up time of 7.7 (IQR: 5.2, 10.7) years, 14,264 (incidence rate: 690.3 per 100,000 person-years) cases of cancers occurred in 2,066,294 person-years, including 2,844 (137.6) cases of lung cancer, 1,048 (103.4 in male participants) cases of prostatic cancer, 1,270 (61.5) cases of colorectal cancer, 1,000 (95.0 in female participants) cases of breast cancer, and 283 (13.7) cases of pancreatic cancer. Details are shown in Table 2 and Supplementary Table S3.

**TABLE 2:** Follow-up and incidence of cancers.

Characteristic	Antihypertensive treatment					
	ACEI	ARB	β-blocker	CCB	TD	Overall
Sample size, N	11,502	98,278	36,162	107,687	16,691	270,320
Follow-up years, median (IQR)	8.4 (5.3 11.8)	7.7 (5.3 10.3)	7.0 (4.8 10.4)	7.6 (5.1 10.7)	9.6 (6.2 11.8)	7.7 (5.2 10.7)
Overall person-years	92,946	747,988	264,606	818,056	142,698	2,066,294
Case of cancer, N/incidence rate, per 100,000 person-years						
Overall cancers	645/694	4,978/665.5	1851/699.5	5,781/706.7	1,009/707.1	14,264/690.3
Lung cancer	123/132.3	1,002/134	400/151.2	1,148/140.3	171/119.8	2,844/137.6
Prostatic cancer^a^	42/88	380/100.5	108/96.5	442/106.8	76/122.9	1,048/103.4
Colorectal cancer	56/60.3	481/64.3	145/54.8	497/60.8	91/63.8	1,270/61.5
Breast cancer^a^	38/84.0	347/93.8	158/103.5	385/95.2	72/89.1	1,000/95
Pancreatic cancer	14/15.1	105/14	27/10.2	111/13.6	26/18.2	283/13.7
Lip, oral, and pharynx cancers	15/16.1	111/14.8	35/13.2	136/16.6	26/18.2	323/15.6
Gastrointestinal cancer	257/276.5	1781/238.1	583/220.3	1997/244.1	393/275.4	5,011/242.5
Respiratory neoplasms	132/142	1,061/141.8	425/160.6	1,230/150.4	176/123.3	3,024/146.3
Skin cancer	4/4.3	29/3.9	4/1.5	33/4	11/7.7	81/3.9
Female reproductive cancer^a^	26/57.5	172/46.5	63/41.3	238/58.9	46/56.9	545/51.8
Male reproductive cancer^a^	43/90.1	387/102.4	111/99.1	449/108.5	77/124.5	1,067/105.3
Urologic cancer	33/35.5	279/37.3	84/31.7	303/37	39/27.3	738/35.7
Endocrine cancer	44/47.3	377/50.4	178/67.3	453/55.4	70/49.1	1,122/54.3
Leukemia	31/33.4	282/37.7	139/52.5	363/44.4	63/44.1	878/42.5

TD, thiazide diuretic; ACEI, angiotensin-converting enzyme inhibitor; ARB, angiotensin II receptor blocker; CCB, calcium channel blocker.

^a^ Incidence was calculated by sex.

**FIGURE 1:**
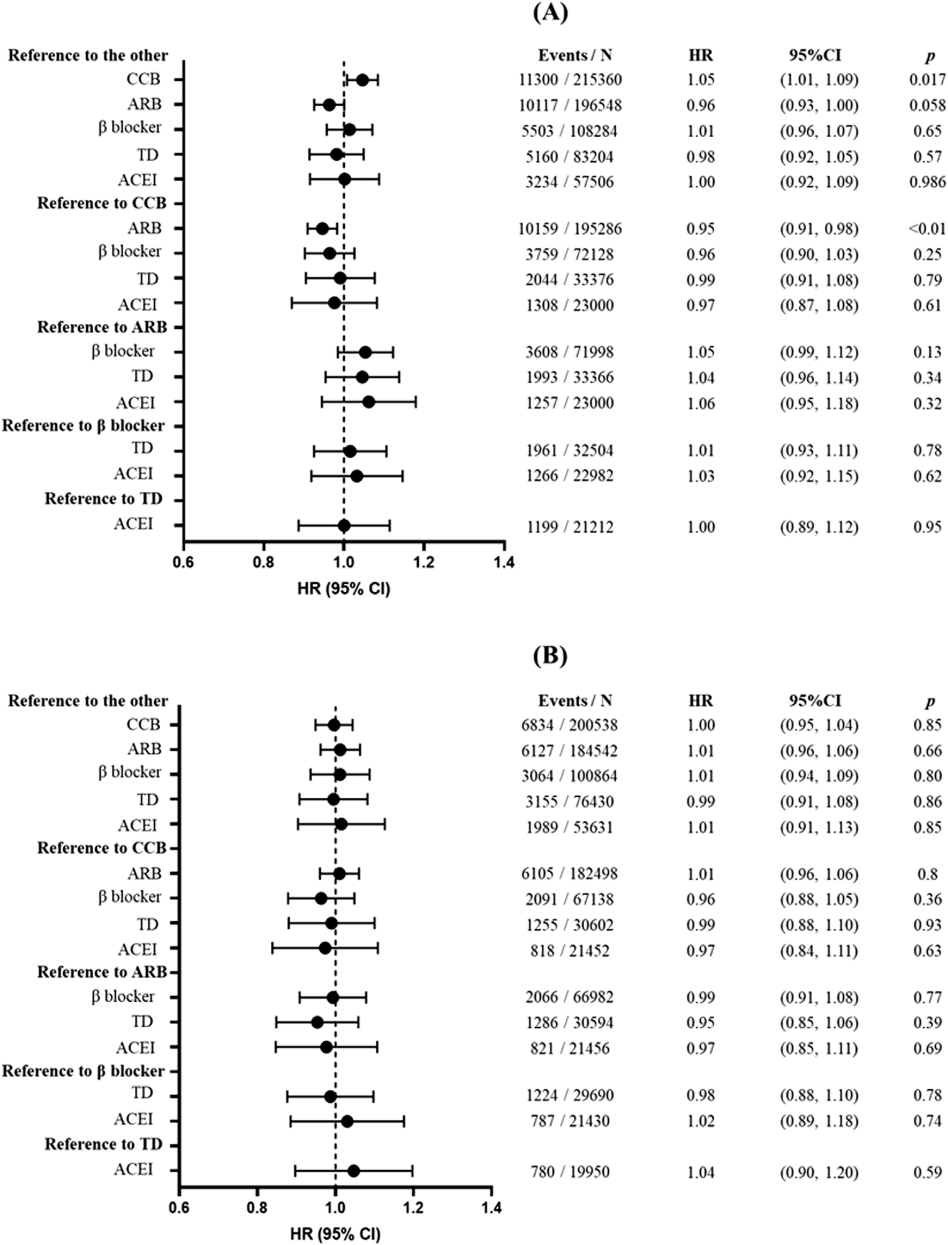
Association between antihypertensive treatments and risk of overall cancers. **(A)** The main analysis without the induction period. **(B)** When performing sensitivity analysis, participants with less than 3 years of follow-up were excluded. Forest plot illustrating hazard ratios (HRs) and corresponding 95% confidence intervals (CIs). Each circle represents the point estimate of HR, with horizontal error bars indicating the 95% confidence interval. The vertical line at HR = 1 serves as the reference. Abbreviations: CCB, calcium channel blocker; ARB, angiotensin receptor blocker; TD, thiazide diuretic; ACEI, angiotensin-converting enzyme inhibitor.

Figure 1 shows the associations between antihypertensive treatments and risk of overall cancers. Compared with the other antihypertensive agents after propensity score matching, CCB was associated with increased cancer risk (HR = 1.05; 95% CI: 1.01, 1.09; *p* = 0.017), whereas marginal significance was shown in association between ARB and a lower cancer risk (HR = 0.96; 95% CI: 0.93, 1.00; *p* = 0.058). β-Blocker, TD, and ACEI showed no significant association with cancer risk (*p* > 0.05). Then, we compared the individual drugs with each other, and only ARB had a significantly lower risk of cancer than CCB (HR = 0.95; 95% CI: 0.91, 0.98; *p* < 0.01). However, all associations of antihypertensive agents (including CCB) with overall cancer risk showed no significance in sensitivity analyses when excluding participants who were followed up for less than 1 year (HR = 1.01; 95% CI: 0.97, 1.05; *p* = 0.78), 2 years (HR = 1.02; 95% CI: 0.98, 1.07; *p* = 0.33), or 3 years (HR = 1.00; 95% CI: 0.95, 1.04; *p* = 0.85) (Supplementary Table S8; Supplementary Table S9; Figure 1B).

**FIGURE 2:**
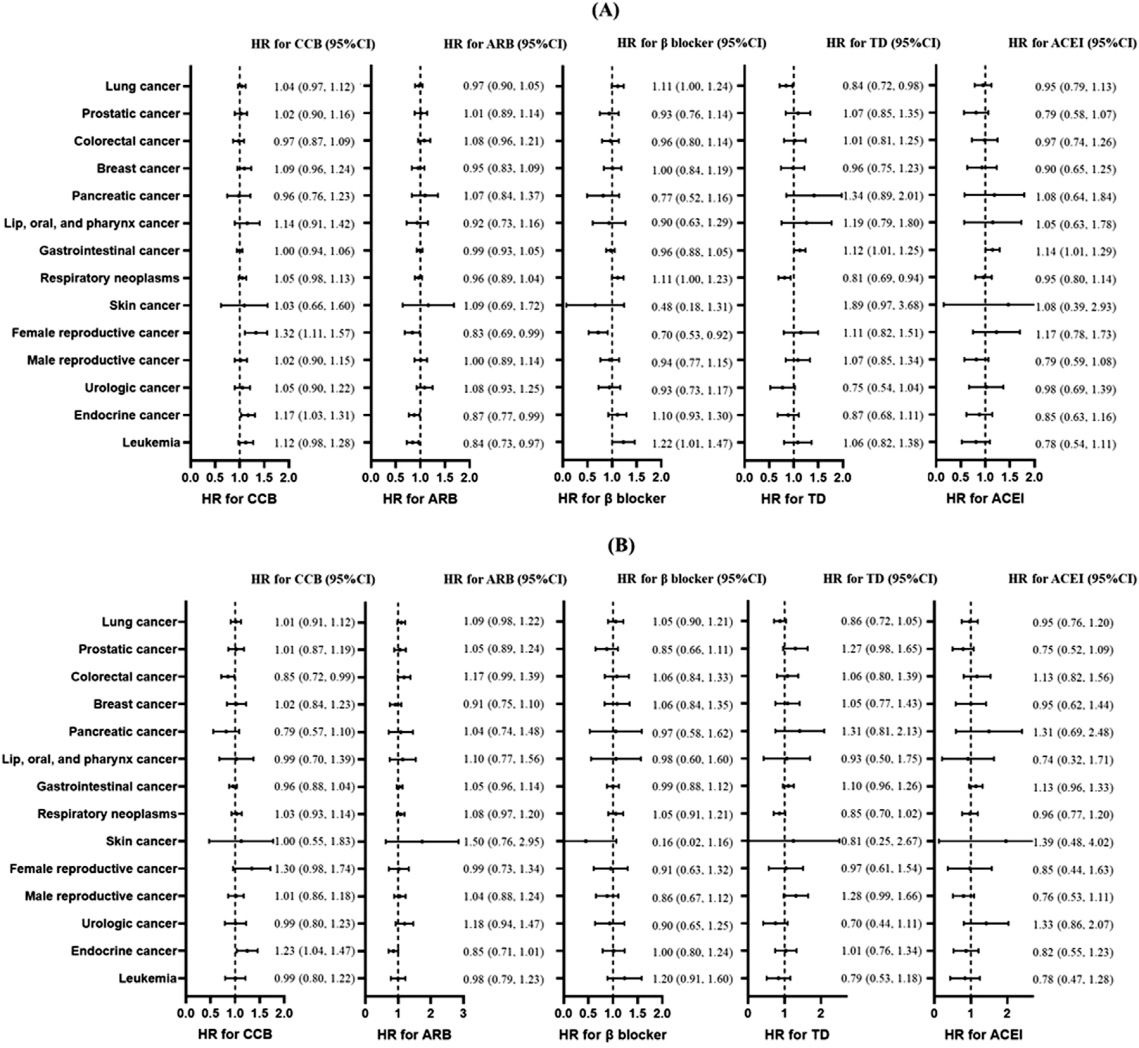
Association between antihypertensive treatments and risk of different types of cancers. **(A)** The main analysis without the induction period. **(B)** When performing sensitivity analysis, participants with less than 3 years of follow-up were excluded. Plots of risk of skin cancer in TD use was not shown because of the excessively wide confidence interval. Forest plot illustrating hazard ratios (HRs) and corresponding 95% confidence intervals (CIs). Each circle represents the point estimate of HR, with horizontal error bars indicating the 95% confidence interval. The vertical line at HR = 1 serves as the reference. Abbreviations: CCB, calcium channel blocker; ARB, angiotensin receptor blocker; TD, thiazide diuretic; ACEI, angiotensin-converting enzyme inhibitor.

After applying PSM, baseline characteristics were well balanced between the groups (Supplementary Figures S1–S5). The associations between antihypertensive agents and specific cancer risk are shown in Figure 2. The CCB was associated with higher risks of reproductive cancers in female individuals (HR = 1.32; 95% CI: 1.11, 1.57; *p* < 0.01) and endocrine cancers (HR = 1.17; 95% CI: 1.03, 1.31; *p* = 0.013); ARB was associated with lower risks of reproductive cancers in female individuals (HR = 0.83; 95% CI: 0.69, 0.99; *p* = 0.043), endocrine cancers (HR = 0.87; 95% CI: 0.77, 0.99; *p* = 0.032), and leukemia (HR = 0.84; 95% CI: 0.73, 0.97; *p* = 0.016); β-blocker was associated with leukemia (HR = 1.22; 95% CI: 1.01, 1.47; *p* = 0.040) and with a lower risk of reproductive cancers in female individuals (HR = 0.70; 95% CI: 0.53, 0.92; *p* = 0.010); TD was associated with a higher risk of gastrointestinal cancer (HR = 1.12; 95% CI: 1.01, 1.25; *p* = 0.032) and lower risks of lung cancer (HR = 0.84; 95% CI: 0.72, 0.98; *p* = 0.026) and respiratory neoplasms (HR = 0.81; 95% CI: 0.69, 0.94; *p* < 0.01); and ACEI was associated with a higher risk of gastrointestinal cancer (HR = 1.14; 95% CI: 1.01, 1.29; *p* = 0.038). However, after excluding participants who developed cancer events within 3 years of follow-up to reduce the influence of reverse causation, only participants with the association between CCB use and a higher risk of endocrine cancer remained (HR = 1.23; 95% CI: 1.04, 1.47; *p* = 0.017).

In subgroup analyses grouped by sex and age, similar results with the main analyses were found. In patients aged over 60 years, β-blocker use had a lower risk of overall cancers (HR = 0.93; 95% CI: 0.86, 1.00; *p* = 0.045). In the subgroup analyses of patients with CCI ≥3, ARB use was associated with a lower risk of cancer (HR = 0.81; 95% CI: 0.73, 0.89; *p* < 0.001), whereas β-blocker use showed a modest but statistically significant association with increased cancer risk (HR = 1.17; 95% CI: 1.03, 1.33; *p* = 0.015) (Supplementary Table S4). Results from sensitivity analyses were also consistent with those from the main analyses (Supplementary Tables S5–S7; Supplementary Tables S10–S12); however, associations of ARB and CCB were no longer significant after excluding participants with less than 1, 2, or 3 years of follow-up (Supplementary Table S8; Supplementary Table S9; Figure 1B). Time-dependent analysis also did not find a significant association between CCB and cancer risk (HR = 0.97; 95% CI: 0.94, 1.00; *p* = 0.056) (Supplementary Table S13). After adjusting for baseline gynecologic hormone medication use among female participants, none of the five classes of antihypertensive drugs showed a significant association with breast cancer risk (Supplementary Table S14). In sensitivity analysis 10, we observed only a marginally significant association between ACE inhibitors and gastric cancer (HR: 1.27, 95% CI: 1.02–1.59, p = 0.033), which remained consistent (HR: 1.27, 95% CI: 1.02–1.59, p = 0.033) after adjusting for baseline *Helicobacter pylori* infection status among participants.

## Discussions

In this large real-world cohort study, 0.27 million patients with hypertension were followed up for a median time of 7.7 years, and 14,264 cases of cancer occurred. In this study, cancer incidence was 690.3 per 100,000 person-years, which is higher than that reported in the previous study (Qiu et al., 2021). This is mainly because the participants in this study were with hypertension and their average age was much older. After controlling for reverse causality, there was no significant association between any antihypertensive agent and overall cancer risk using the PSM method which matched for age, sex, smoking status, drinking status, sports frequency, BMI, CCI, dyslipidemia history, depression history, arthritis history, lipid-regulating agent use, antidiabetic agent use, NSAID use, corticoid agent use, anticoagulant use, antiplatelet agent use, healthcare utilization, baseline blood pressure level, the time from initial hypertension diagnosis to the initiation of antihypertensive medication, adherence, and the year of the index date.

Our findings were consistent with several previous studies. An individual-based meta-analysis (Copland et al., 2021) found that there was no significant association of ACEI, ARB, β-blocker, and TD with cancer risk, whether compared with the placebo or with the other antihypertensive medications. In 4.2 years (median) of follow-up, CCB use had an increased risk for cancer compared with the other antihypertensive agents (HR = 1.06; 95% CI: 1.01, 1.11) but not with the placebo (HR = 1.00; 95% CI: 0.90, 1.10). Recently, a population-based cohort study (Wang et al., 2023) with 101,370 participants found that CCB use was associated with an increased risk of total cancer (HR = 1.11; 95% CI: 1.05, 1.18) during a mean follow-up of 5.1 years. However, potential residual confounding might have influenced the results of that study, such as the absence of medication history, healthcare utilization, and the year of the enrollment. Furthermore, undiagnosed cancers may systematically influence antihypertensive selection in patients with hypertension as clinicians may avoid hepatically metabolized drugs in liver metastasis or discontinue renally excreted agents during nephrotoxic chemotherapy. This protopathic bias could lead to deviation from the null hypothesis in pharmacoepidemiologic analyses. In such scenarios, extending the washout period or excluding patients diagnosed with cancer within few years of antihypertensive initiation would help mitigate this bias. In sensitivity analysis of the previous study, excluding cancer cases who were diagnosed within 2 years of enrollment, the association between CCB and cancer risk tends to be conservative (HR: 1.07; 95% CI: 1.00, 1.14). Similarly, in our study, the main analysis incorporating a 4-year washout period found no significant association between ACEI, ARB, β-blocker, or TD and cancer risk; however, CCB showed a potential marginal association (HR = 1.05; 95% CI: 1.01, 1.09; *p* = 0.017). However, after excluding enrolled participants who were followed up for less than 1 year/2 years/3 years to minimize the influence of reverse causation, the association between CCB and cancer risk disappeared (HR for CCB after excluding participants who developed cancer within 3 years = 1.01; 95% CI: 0.96, 1.06; *p* = 0.80). Although the acute risk could also explain this result, there was no evidence that showed an acute increase in cancer risk associated with short-term CCB use, and RCT studies found no significant association between CCB and cancer risk in 1, 2, or 3 years (Copland et al., 2021). This implies that reverse causality might exist in real-world settings, and an induction period is necessary to be established to reduce the bias.

As for specific cancers, this study mainly focused on lung cancer, prostatic cancer, colorectal cancer, breast cancer, and pancreatic cancer, which were reported to have potential associations with antihypertensive medication. In this study, we found no significant association in these five cancers.

We found that CCB use was associated with an increased risk of endocrine cancers, in particular thyroid cancer (1,106 cases from 1,122 cases of endocrine cancers). Biologically, up to 70% of thyroid carcinomas are caused by mutations that activate the RAS/ERK mitogenic signaling pathway (Zaballos et al., 2019; Park et al., 2023; Xing, 2013). Although CCB inhibited ERK signaling in pancreatic cancer (Principe et al., 2022) and ovarian cancer (Lee et al., 2020) and activated ERK signaling in MDA-MB-231 cells from breast carcinoma (Zhao et al., 2017), there is no evidence that CCB has an effect on ERK signaling in thyroid carcinomas. Furthermore, biomedical study is needed to ensure the effect of CCB on the ERK pathway in thyroid cancer.

Although some international guidelines have removed β-blockers from their first-line antihypertensive recommendations, authoritative Chinese guidelines (Liu, 2020; Wang, 2025) continue to maintain β-blockers as a first-line treatment option, particularly for patients with comorbid conditions such as heart failure or prior myocardial infarction. In our study cohort, patients who were prescribed β-blockers demonstrated higher baseline CCI scores, along with elevated prevalence of dyslipidemia, depression, and osteoarthritis—clinical characteristics that might theoretically predispose to increased cancer risk. However, after implementing comprehensive PSM to account for these potential confounders, our analysis indicated no statistically significant association between β-blocker use and subsequent cancer risk.

Attention should also be paid to the skin cancer risk associated with TD. The incidence of skin cancer in East Asia is much lower than that in Europe (Lin et al., 2021). Due to the low incidence of skin cancer in China, in this study, we found no significant associations between antihypertensive agents and TD. Several antihypertensive drugs, including TD and CCB, were considered agents with drug-induced photosensitivity, which lead to potential skin cancer risk through the interaction between a chemical agent and UV radiation (Gandini et al., 2018; Hou et al., 2024). Previous studies showed that thiazide and thiazide-like diuretics have photosensitizing potential, but evidence regarding the association between TD use and skin cancer is still lacking due to important methodological limitations (Kreutz et al., 2019). The use of CCB was reported to be associated with more severe actinic keratosis (Gioppo et al., 2023), whereas evidence regarding the association between CCB use and the risk of skin cancer is still controversial.

Our research provides new evidence for the long-term association between antihypertensive agent use and cancer risk in a large population in real-world settings. To mimic effectiveness of RCTs, it is important to control potential confounding in real-world studies. In this study, PSM and IPW were used to reduce the bias caused by baseline difference between groups. We also considered and controlled time-related bias, such as immortal time bias and time of the index date. One of the strengths of our study is that the associations between antihypertensive medications and the risk of different specific cancers were estimated separately. Moreover, we used multiple methods to control for potential confounding, including PSM, inverse-probability weighting, subgroup analyses, and 10 sensitivity analyses.

This study also has several limitations. Combinations of antihypertensive medications were also recommended to be used in high-risk patients in guidelines (Liu, 2020; Wang, 2025). Considering potential indication bias, patients on initial combination therapy were not included in this study. Furthermore, research is needed to compare the cancer risk between different combination antihypertensive agents and between single therapy and combination therapy when bias could be well controlled. Second, actual situation of treatment might not be consistent with the prescriptions, which is a common drawback of observational pharmacoepidemiologic studies. We attempted to control for this confounding by using antihypertensive treatment frequency to indicate actual adherence. Moreover, it is noteworthy that the cancer risk associated with the use of antihypertensive medications varies among patients with diverse genetic backgrounds (Zheng et al., 2024). Furthermore, studies that consider the detailed genetic background are still needed.

## Conclusion

Although previous studies suggested a potential cancer risk with CCB use, and the association between hypertensive agents and different cancers remained controversial, in this study, we found no clinically meaningful cancer risk associated with antihypertensive medications. However, the association between CCB and specific cancer still requires further research. These findings should be interpreted with caution due to the potential residual confounding.

## Data Availability

The data analyzed in this study are subjected to the following licenses/restrictions: The datasets generated and/or analyzed during the current study are not publicly available due to policy reasons but are available from the corresponding author on reasonable request. Requests to access these datasets should be directed to qywu118@163.com.
