# Supplementary material for: Antihypertensive medications and cancer risk: Evidence from 0.27 million patients with newly diagnosed hypertension
Source: Front Pharmacol. 2025 Jul 1;16:1559604. doi: 10.3389/fphar.2025.1559604 (PMC12259635; doi:10.3389/fphar.2025.1559604)
Supplement: Supplementary file 1 [file Supplementaryfile1.docx]

**Abbreviations.** TD, Thiazide diuretics; ACEI, Angiotensin-Converting Enzyme Inhibitors; ARB, Angiotensin Receptor Blockers; CCB, Calcium Channel Blockers; BB, β-blocker; HR, hazard ratio; CI, confidence interval.

Supplementary Table 1. Charlson Comorbidity Index (CCI) Components and Weight.

| **Condition** | **Weight** | **ICD-10 Codes (Example)** |
| --- | --- | --- |
| Myocardial infarction | 1 | I21, I22, I25.2 |
| Congestive heart failure | 1 | I50 |
| Peripheral vascular disease | 1 | I73, I70.2, I79.2 |
| Cerebrovascular disease | 1 | I60-I69 |
| Dementia | 1 | F00-F03, F05.1, G30 |
| Chronic pulmonary disease | 1 | J40-J47, J60-J67 |
| Rheumatologic disease | 1 | M05, M06, M32, M34 |
| Peptic ulcer disease | 1 | K25-K28 |
| Mild liver disease | 1 | K70, K73, K74, K76.0 |
| Diabetes without complications | 1 | E10.9, E11.9, E14.9 |
| Diabetes with complications | 2 | E10.2-E10.8, E11.2-E11.8 |
| Hemiplegia or paraplegia | 2 | G81, G82 |
| Renal disease | 2 | N03, N05, N18, N19 |
| Any malignancy (except skin) | 2 | C00-C26, C30-C34, C37-C41, C43, C45-C58, C60-C76, C81-C85, C88, C90, C96 |
| Moderate/severe liver disease | 3 | K70.4, K71.1, K72, K76.6, I85 |
| Metastatic solid tumor | 6 | C77-C80 |
| AIDS/HIV | 6 | B20-B24 |

Supplementary Table 2. Extended baseline characteristics at the index date.

| **Characteristics** | **TD**  (N=16691) | **ACEI**  (N=11502) | **ARB**  (N=98278) | **β Blocker**  (N=36162) | **CCB**  (N=107687) | **Overall**  (N=270320) |
| --- | --- | --- | --- | --- | --- | --- |
| Year, N (%) |  |  |  |  |  |  |
| 2009 | 2423 (14.5) | 2051 (17.8) | 9202 (9.4) | 2994 (8.3) | 12135 (11.3) | 28805 (10.7) |
| 2010 | 3000 (18.0) | 1701 (14.8) | 10907 (11.1) | 2990 (8.3) | 12646 (11.7) | 31244 (11.6) |
| 2011 | 3076 (18.4) | 1367 (11.9) | 10660 (10.8) | 5074 (14.0) | 12011 (11.2) | 32188 (11.9) |
| 2012 | 1697 (10.2) | 862 (7.5) | 9999 (10.2) | 2880 (8.0) | 9905 (9.2) | 25343 (9.4) |
| 2013 | 1453 (8.7) | 754 (6.6) | 9583 (9.8) | 2644 (7.3) | 8893 (8.3) | 23327 (8.6) |
| 2014 | 1037 (6.2) | 778 (6.8) | 8797 (9.0) | 2673 (7.4) | 8859 (8.2) | 221448.2) |
| 2015 | 978 (5.9) | 632 (5.5) | 7910 (8.0) | 2585 (7.1) | 7878 (7.3) | 19983 (7.4) |
| 2016 | 1076 (6.4) | 889 (7.7) | 8880 (9.0) | 3069 (8.5) | 9342 (8.7) | 23256 (8.6) |
| 2017 | 883 (5.3) | 968 (8.4) | 10079 (10.3) | 4601 (12.7) | 10905 (10.1) | 27436 (10.1) |
| 2018 | 593 (3.6) | 739 (6.4) | 6317 (6.4) | 3243 (9.0) | 7414 (6.9) | 18306 (6.8) |
| 2019 | 475 (2.8) | 761 (6.6) | 5944 (6.0) | 3409 (9.4) | 7699 (7.1) | 18288 (6.8) |

Supplementary Table 3. Incidence of cancers and 95% its confidence interval.

|  | **Antihypertensive treatments** | | | | |  |
| --- | --- | --- | --- | --- | --- | --- |
|  | **ACEI** | **ARB** | **β blocker** | **CCB** | **TD** | **Overall** |
| Overall cancers | 694 (640.4, 747.5) | 665.5 (647, 684) | 699.5 (667.7, 731.4) | 706.7 (688.5, 724.9) | 707.1 (663.5, 750.7) | 690.3 (679, 701.6) |
| Lung cancer | 132.3 (108.9, 155.7) | 134 (125.7, 142.3) | 151.2 (136.4, 166) | 140.3 (132.2, 148.5) | 119.8 (101.9, 137.8) | 137.6 (132.6, 142.7) |
| Prostatic cancer * | 88 (63, 117.7) | 100.5 (45.7, 55.9) | 96.5 (33.1, 48.5) | 106.8 (49, 59.1) | 122.9 (41.3, 65.2) | 103.4 (47.6, 53.8) |
| Colorectal cancer | 60.3 (44.5, 76) | 64.3 (58.6, 70.1) | 54.8 (45.9, 63.7) | 60.8 (55.4, 66.1) | 63.8 (50.7, 76.9) | 61.5 (58.1, 64.8) |
| Breast cancer * | 84 (55.8, 107.8) | 93.8 (41.5, 51.3) | 103.5 (50.4, 69) | 95.2 (42.4, 51.8) | 89.1 (38.8, 62.1) | 95 (45.4, 51.4) |
| Pancreatic cancer | 15.1 (7.2, 23) | 14 (11.4, 16.7) | 10.2 (6.4, 14.1) | 13.6 (11, 16.1) | 18.2 (11.2, 25.2) | 13.7 (12.1, 15.3) |
| Lip, oral, and pharynx cancer | 16.1 (8, 24.3) | 14.8 (12.1, 17.6) | 13.2 (8.8, 17.6) | 16.6 (13.8, 19.4) | 18.2 (11.2, 25.2) | 15.6 (13.9, 17.3) |
| Gastrointestinal cancer | 276.5 (242.7, 310.3) | 238.1 (227, 249.2) | 220.3 (202.4, 238.2) | 244.1 (233.4, 254.8) | 275.4 (248.2, 302.6) | 242.5 (235.8, 249.2) |
| Respiratory neoplasms | 142 (117.8, 166.2) | 141.8 (133.3, 150.4) | 160.6 (145.3, 175.9) | 150.4 (142, 158.8) | 123.3 (105.1, 141.6) | 146.3 (141.1, 151.6) |
| Skin cancer | 4.3 (0.1, 8.5) | 3.9 (2.5, 5.3) | 1.5 (0, 3) | 4 (2.7, 5.4) | 7.7 (3.2, 12.3) | 3.9 (3.1, 4.8) |
| Female reproductive cancer * | 57.5 (34.4, 77.5) | 46.5 (19.6, 26.4) | 41.3 (17.9, 29.7) | 58.9 (25.4, 32.8) | 56.9 (22.9, 41.6) | 51.8 (24.2, 28.6) |
| Male reproductive cancer * | 90.1 (64.9, 120.2) | 102.4 (46.6, 56.9) | 99.1 (34.1, 49.8) | 108.5 (49.8, 60) | 124.5 (41.9, 66) | 105.3 (48.5, 54.7) |
| Urologic cancer | 35.5 (23.4, 47.6) | 37.3 (32.9, 41.7) | 31.7 (25, 38.5) | 37 (32.9, 41.2) | 27.3 (18.8, 35.9) | 35.7 (33.1, 38.3) |
| Endocrine cancer | 47.3 (33.4, 61.3) | 50.4 (45.3, 55.5) | 67.3 (57.4, 77.2) | 55.4 (50.3, 60.5) | 49.1 (37.6, 60.5) | 54.3 (51.1, 57.5) |
| Leukemia | 33.4 (21.6, 45.1) | 37.7 (33.3, 42.1) | 52.5 (43.8, 61.3) | 44.4 (39.8, 48.9) | 44.1 (33.2, 55.1) | 42.5 (39.7, 45.3) |

Supplementary table 4. Subgroup analysis of association between antihypertensive medications and cancer risk by age and sex.

|  | N | Events | HR | -95% CI | +95% CI | *p* |
| --- | --- | --- | --- | --- | --- | --- |
| < 60 years old |  |  |  |  |  |  |
| CCB | 136282 | 5343 | 1.10 | 1.05 | 1.16 | <0.001 |
| ARB | 131224 | 5347 | 0.92 | 0.87 | 0.97 | <0.01 |
| BB | 68500 | 2560 | 1.03 | 0.95 | 1.11 | 0.53 |
| TD | 48568 | 2170 | 1.07 | 0.96 | 1.18 | 0.21 |
| ACEI | 37863 | 1661 | 0.94 | 0.83 | 1.06 | 0.33 |
| ≥ 60 years old |  |  |  |  |  |  |
| CCB | 79046 | 5792 | 1.06 | 1.01 | 1.11 | 0.033 |
| ARB | 65326 | 4818 | 1.00 | 0.95 | 1.06 | 0.965 |
| BB | 39614 | 3062 | 0.93 | 0.86 | 1.00 | 0.045 |
| TD | 34483 | 2826 | 0.99 | 0.90 | 1.09 | 0.84 |
| ACEI | 19624 | 1565 | 1.07 | 0.94 | 1.21 | 0.30 |
| Male |  |  |  |  |  |  |
| CCB | 110220 | 6601 | 1.05 | 1.00 | 1.10 | 0.058 |
| ARB | 99922 | 5889 | 0.97 | 0.92 | 1.02 | 0.20 |
| BB | 46626 | 2808 | 0.94 | 0.87 | 1.02 | 0.15 |
| TD | 36894 | 2884 | 0.99 | 0.90 | 1.08 | 0.80 |
| ACEI | 30252 | 1955 | 1.01 | 0.91 | 1.13 | 0.81 |
| Female |  |  |  |  |  |  |
| CCB | 101106 | 4651 | 1.06 | 1.00 | 1.12 | 0.044 |
| ARB | 96630 | 4262 | 0.94 | 0.89 | 1.00 | 0.058 |
| BB | 61530 | 2861 | 0.98 | 0.91 | 1.06 | 0.65 |
| TD | 46206 | 2354 | 0.93 | 0.85 | 1.03 | 0.14 |
| ACEI | 27220 | 1256 | 0.98 | 0.86 | 1.13 | 0.82 |
| CCI<3 |  |  |  |  |  |  |
| CCB | 202529 | 9836 | 1.04 | 1.00 | 1.08 | 0.083 |
| ARB | 181250 | 8414 | 0.99 | 0.95 | 1.03 | 0.68 |
| BB | 98680 | 4469 | 0.98 | 0.92 | 1.05 | 0.58 |
| TD | 78380 | 4548 | 0.97 | 0.90 | 1.04 | 0.35 |
| ACEI | 53301 | 2722 | 1.00 | 0.91 | 1.10 | 0.93 |
| CCI≥3 |  |  |  |  |  |  |
| CCB | 12831 | 1464 | 1.10 | 1.00 | 1.22 | 0.061 |
| ARB | 15298 | 1703 | 0.81 | 0.73 | 0.89 | <0.001 |
| BB | 9604 | 1034 | 1.17 | 1.03 | 1.33 | 0.015 |
| TD | 4824 | 612 | 1.14 | 0.94 | 1.39 | 0.19 |
| ACEI | 4205 | 512 | 1.01 | 0.81 | 1.26 | 0.952 |

Supplementary table 5. Sensitivity analysis in patients who persisted single antihypertensive treatment without change during the follow-up period.

| Agents | Reference | N | Events | HR | -95% CI | +95% CI | *p* |
| --- | --- | --- | --- | --- | --- | --- | --- |
| ARB | CCB | 77392 | 3046 | 0.89 | 0.83 | 0.96 | <0.01 |
| BB | CCB | 37208 | 1575 | 0.81 | 0.73 | 0.89 | <0.001 |
| TD | CCB | 12804 | 682 | 0.80 | 0.69 | 0.93 | <0.01 |
| ACEI | CCB | 7228 | 334 | 0.65 | 0.52 | 0.81 | <0.001 |
| BB | ARB | 37274 | 1442 | 0.97 | 0.87 | 1.07 | 0.50 |
| TD | ARB | 12632 | 653 | 0.84 | 0.72 | 0.98 | 0.028 |
| ACEI | ARB | 7234 | 288 | 0.86 | 0.68 | 1.08 | 0.20 |
| TD | BB | 12512 | 614 | 0.95 | 0.81 | 1.11 | 0.51 |
| ACEI | BB | 7200 | 285 | 0.84 | 0.66 | 1.06 | 0.13 |
| ACEI | TD | 6716 | 258 | 0.96 | 0.76 | 1.23 | 0.77 |

Supplementary table 6. Sensitivity analysis in patients with hypertension diagnosed by ICD-10.

| Agents | Reference | N | Events | HR | -95% CI | +95% CI | *p* |
| --- | --- | --- | --- | --- | --- | --- | --- |
| ARB | CCB | 188878 | 10028 | 0.96 | 0.92 | 0.99 | 0.026 |
| BB | CCB | 48648 | 3099 | 0.91 | 0.84 | 0.97 | <0.01 |
| TD | CCB | 26820 | 1767 | 0.92 | 0.84 | 1.01 | 0.077 |
| ACEI | CCB | 21758 | 1293 | 0.96 | 0.86 | 1.08 | 0.50 |
| BB | ARB | 48658 | 3031 | 0.95 | 0.89 | 1.02 | 0.16 |
| TD | ARB | 26808 | 1738 | 0.94 | 0.86 | 1.04 | 0.23 |
| ACEI | ARB | 21756 | 1246 | 1.01 | 0.91 | 1.13 | 0.80 |
| TD | BB | 25838 | 1667 | 0.98 | 0.89 | 1.08 | 0.68 |
| ACEI | BB | 21684 | 1221 | 1.07 | 0.96 | 1.20 | 0.23 |
| ACEI | TD | 18576 | 1136 | 1.00 | 0.89 | 1.13 | 0.96 |

Supplementary table 7. Sensitivity analysis in participants who started to use antihypertensive medication within 1 year after the first diagnosis of hypertension.

| Agents | Reference | N | Events | HR | -95% CI | +95% CI | *p* |
| --- | --- | --- | --- | --- | --- | --- | --- |
| ARB | CCB | 144902 | 6582 | 0.94 | 0.89 | 0.98 | <0.01 |
| BB | CCB | 57920 | 2705 | 0.96 | 0.89 | 1.04 | 0.35 |
| TD | CCB | 25346 | 1449 | 0.92 | 0.83 | 1.02 | 0.093 |
| ACEI | CCB | 15964 | 776 | 0.90 | 0.78 | 1.04 | 0.16 |
| BB | ARB | 57810 | 2590 | 1.05 | 0.97 | 1.13 | 0.22 |
| TD | ARB | 25334 | 1416 | 0.96 | 0.86 | 1.06 | 0.40 |
| ACEI | ARB | 15974 | 756 | 0.96 | 0.83 | 1.10 | 0.53 |
| TD | BB | 24200 | 1379 | 0.93 | 0.83 | 1.03 | 0.16 |
| ACEI | BB | 15976 | 764 | 0.93 | 0.81 | 1.07 | 0.31 |
| ACEI | TD | 14608 | 683 | 1.03 | 0.89 | 1.20 | 0.66 |

Supplementary table 8. Sensitivity analysis – Participants with less than 1 years of follow-up were excluded.

| Agents | Reference | N | Events | HR | -95% CI | +95% CI | *p* |
| --- | --- | --- | --- | --- | --- | --- | --- |
| ARB | CCB | 190326 | 8088 | 1.00 | 0.95 | 1.04 | 0.89 |
| BB | CCB | 69954 | 2797 | 1.00 | 0.93 | 1.08 | 0.959 |
| TD | CCB | 31804 | 1607 | 0.94 | 0.85 | 1.03 | 0.20 |
| ACEI | CCB | 22418 | 1037 | 0.99 | 0.88 | 1.12 | 0.93 |
| BB | ARB | 69828 | 2816 | 0.97 | 0.90 | 1.05 | 0.48 |
| TD | ARB | 31794 | 1617 | 0.92 | 0.84 | 1.02 | 0.099 |
| ACEI | ARB | 22422 | 1016 | 1.03 | 0.91 | 1.17 | 0.60 |
| TD | BB | 30896 | 1540 | 0.97 | 0.88 | 1.07 | 0.57 |
| ACEI | BB | 22398 | 1035 | 0.98 | 0.87 | 1.11 | 0.80 |
| ACEI | TD | 20560 | 967 | 1.04 | 0.91 | 1.17 | 0.60 |

Supplementary table 9. Sensitivity analysis – Participants with less than 2 years of follow-up were excluded.

|  | N | Events | HR | -95%CI | +95%CI | *p* |
| --- | --- | --- | --- | --- | --- | --- |
| CCB | 205696 | 7888 | 1.022 | 0.978 | 1.068 | 0.331 |
| ARB | 188630 | 7056 | 1.023 | 0.9761 | 1.072 | 0.344 |
| β blocker | 103066 | 3607 | 0.9908 | 0.9245 | 1.062 | 0.795 |
| THIA | 77937 | 3620 | 0.968 | 0.8917 | 1.051 | 0.438 |
| ACEI | 54954 | 2247 | 1.04 | 0.939 | 1.152 | 0.45 |

Supplementary table 10. Sensitivity analysis in participants who enrolled from 2009 to 2017.

| Agents | Reference | N | Events | HR | -95% CI | +95% CI | *p* |
| --- | --- | --- | --- | --- | --- | --- | --- |
| ARB | CCB | 170712 | 9377 | 0.96 | 0.93 | 1.00 | 0.074 |
| BB | CCB | 58930 | 3361 | 0.93 | 0.87 | 1.00 | 0.043 |
| TD | CCB | 31242 | 1965 | 1.00 | 0.92 | 1.09 | 0.983 |
| ACEI | CCB | 20002 | 1167 | 1.03 | 0.92 | 1.16 | 0.61 |
| BB | ARB | 58904 | 3224 | 1.02 | 0.95 | 1.09 | 0.62 |
| TD | ARB | 31224 | 1917 | 1.04 | 0.95 | 1.14 | 0.35 |
| ACEI | ARB | 20004 | 1183 | 0.99 | 0.89 | 1.11 | 0.91 |
| TD | BB | 30290 | 1932 | 0.98 | 0.89 | 1.07 | 0.63 |
| ACEI | BB | 19982 | 1192 | 0.98 | 0.87 | 1.09 | 0.66 |
| ACEI | TD | 19194 | 1151 | 0.97 | 0.87 | 1.09 | 0.66 |

Supplementary table 11. Sensitivity analysis - Using inverse probability weighting methods instead of PSM for association analysis.

| Agents | Reference | N | Events | HR | -95% CI | +95% CI | *p* |
| --- | --- | --- | --- | --- | --- | --- | --- |
| ARB | CCB | 205965 | 10759 | 0.94 | 0.91 | 0.98 | <0.01 |
| BB | CCB | 143849 | 7632 | 0.96 | 0.90 | 1.01 | 0.11 |
| TD | CCB | 124378 | 6790 | 0.96 | 0.90 | 1.03 | 0.30 |
| ACEI | CCB | 119189 | 6426 | 0.97 | 0.90 | 1.06 | 0.52 |
| BB | ARB | 134440 | 6829 | 1.03 | 0.97 | 1.09 | 0.34 |
| TD | ARB | 114969 | 5987 | 1.03 | 0.96 | 1.11 | 0.45 |
| ACEI | ARB | 109780 | 5623 | 1.03 | 0.95 | 1.12 | 0.47 |
| TD | BB | 52853 | 2860 | 1.02 | 0.94 | 1.11 | 0.62 |
| ACEI | BB | 47664 | 2496 | 1.00 | 0.91 | 1.10 | 0.95 |
| ACEI | TD | 28193 | 1654 | 0.99 | 0.90 | 1.10 | 0.90 |

Supplementary table 12. Sensitivity analysis - competing risk models.

| Agents | Reference | HR | -95% CI | +95% CI | *p* |
| --- | --- | --- | --- | --- | --- |
| ARB | CCB | 0.95 | 0.91 | 0.98 | <0.01 |
| BB | CCB | 0.96 | 0.90 | 1.02 | 0.20 |
| TD | CCB | 0.97 | 0.89 | 1.06 | 0.53 |
| ACEI | CCB | 0.97 | 0.87 | 1.08 | 0.58 |
| BB | ARB | 1.05 | 0.98 | 1.12 | 0.19 |
| TD | ARB | 1.02 | 0.94 | 1.12 | 0.58 |
| ACEI | ARB | 1.05 | 0.94 | 1.18 | 0.36 |
| TD | BB | 1.00 | 0.91 | 1.09 | 0.94 |
| ACEI | BB | 1.03 | 0.93 | 1.16 | 0.54 |
| ACEI | TD | 1.00 | 0.90 | 1.12 | 0.94 |

Supplementary table 13. Sensitivity analysis - Time-dependent analysis.

|  | HR | -95% CI | +95% CI | *p* |
| --- | --- | --- | --- | --- |
| CCB | 0.97 | 0.94 | 1.00 | 0.056 |
| ARB | 1.02 | 0.99 | 1.05 | 0.18 |
| β blocker | 1.05 | 1.00 | 1.10 | 0.033 |
| TD | 1.01 | 0.96 | 1.06 | 0.67 |
| ACEI | 1.06 | 0.98 | 1.14 | 0.15 |

Supplementary table 14. Association of antihypertensive treatments with risk for breast cancer (additionally adjusting for gynecologic hormone medication use).

| **Breast Cancer Risk** | **HR** | **-95% CI** | **+95% CI** | ***p*** |
| --- | --- | --- | --- | --- |
| CCB | 1.08 | 0.93 | 1.25 | 0.30 |
| ARB | 0.90 | 0.77 | 1.35 | 1.04 |
| β blocker | 1.01 | 0.84 | 1.23 | 0.88 |
| TD | 1.07 | 0.82 | 1.40 | 0.60 |
| ACEI | 0.86 | 0.61 | 1.22 | 0.40 |


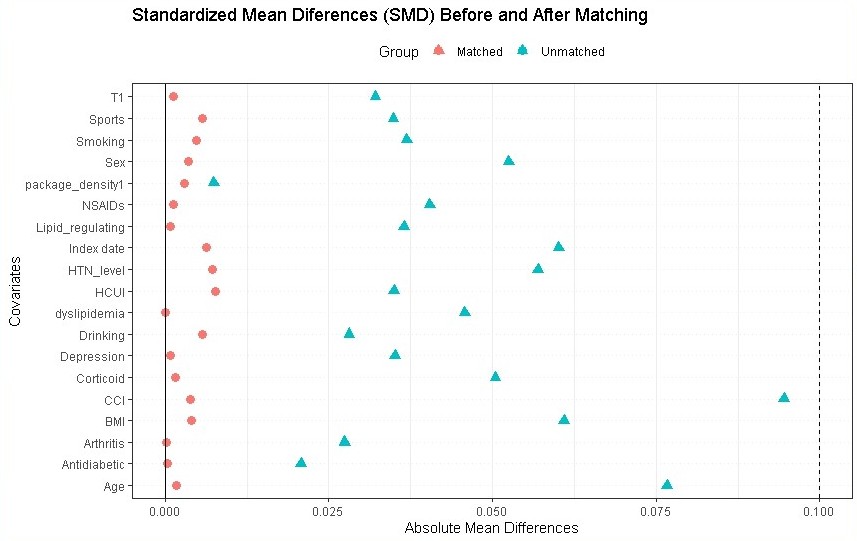


Supplementary Figure 1. Standardized Mean Differences (SMD) Before and After Matching for CCBs.


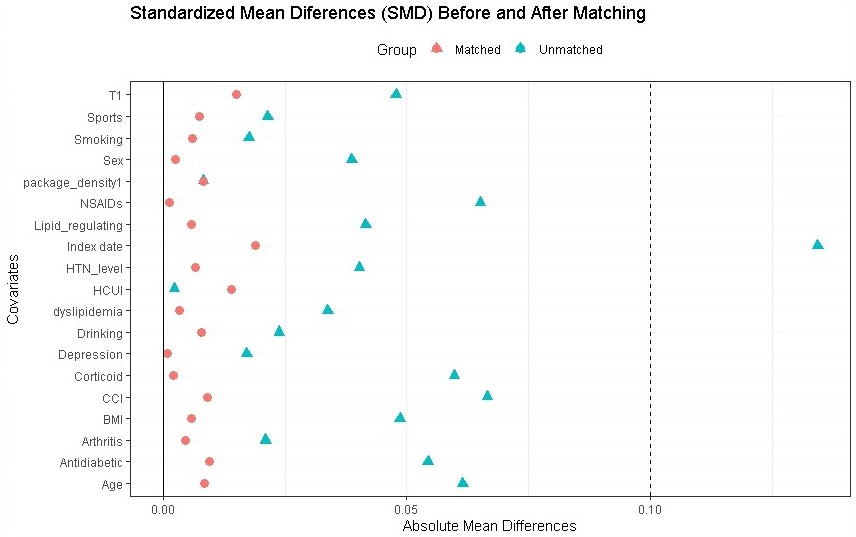


Supplementary Figure 2. Standardized Mean Differences (SMD) Before and After Matching for ARBs.


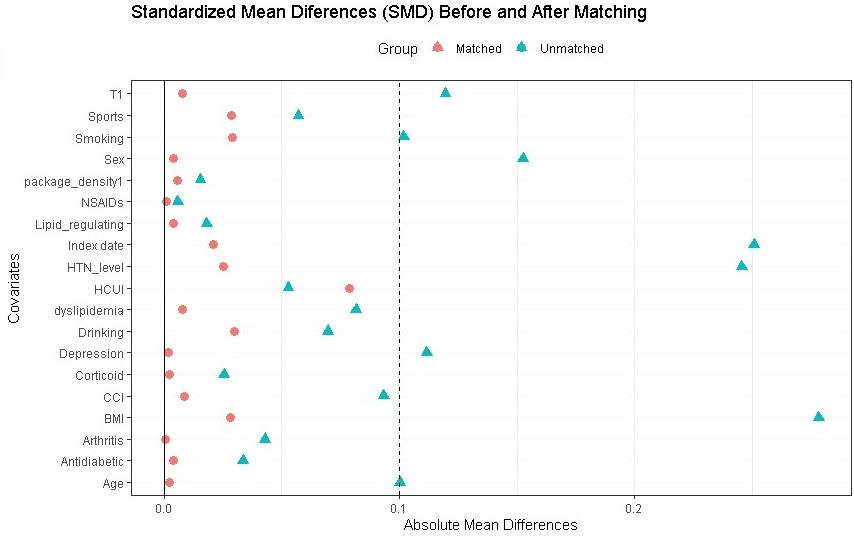


Supplementary Figure 3. Standardized Mean Differences (SMD) Before and After Matching for β blocker.


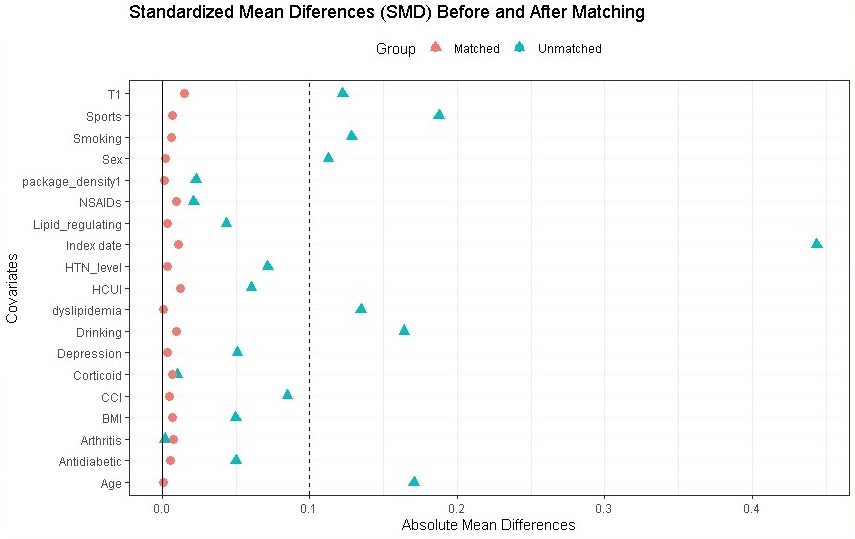


Supplementary Figure 4. Standardized Mean Differences (SMD) Before and After Matching for thiazide diuretic.


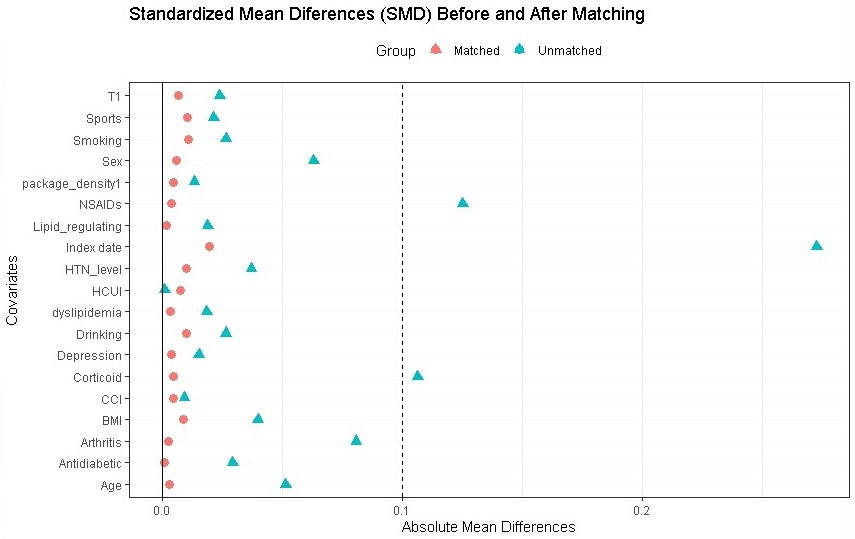


Supplementary Figure 5. Standardized Mean Differences (SMD) Before and After Matching for ACEI.
